# Development and Validation of Comorbidity Severity Adjustment Methods in Mortality Models for Acute Cerebrovascular Disease Using Survival and Machine Learning Analyses

**DOI:** 10.3390/jcm14103281

**Published:** 2025-05-08

**Authors:** Yeaeun Kim, Jongho Park

**Affiliations:** 1 Department of Health Care Management, Catholic University of Pusan, Busan 46252, Republic of Korea; eyeany@cup.ac.kr; 2 Department of Health and Medical Information, Daegu University, Gyeongsan-si 38453, Republic of Korea

**Keywords:** comorbidity-based severity adjustment, machine learning in mortality prediction, acute cerebrovascular disease

## Abstract

**Background/Objectives**: This study aimed to develop and validate comorbidity-based severity adjustment methods for acute cerebrovascular disease by recalibrating the Charlson Comorbidity Index (CCI) and constructing a CCS-based comorbidity index to improve mortality risk prediction. **Methods**: Using the Korea Disease Control Agency’s Discharge Injury In-depth Survey Dataset (2013–2022), we applied Cox proportional hazards regression and machine learning techniques, including LASSO, CART, Random Forests, GBM, and ANN, to recalibrate the CCI and develop a CCS-based comorbidity index. **Results**: The recalibrated Charlson Comorbidity Index (m-CCI) and the newly developed CCS-based comorbidity index (m-CCS) demonstrated improved predictive performance for in-hospital mortality. Among the machine learning models, GBM (AUC = 0.835) and ANN (AUC = 0.830) demonstrated the highest predictive accuracy, with m-CCS consistently outperforming other indices. **Conclusions**: The recalibrated m-CCI and newly developed m-CCS comorbidity indices enhance mortality risk adjustment for acute cerebrovascular disease patients in Korea. The superior performance of machine learning models underscores their potential for enhancing severity adjustment in hospital benchmarking and quality assessment.

## 1. Introduction

Acute cerebrovascular disease is a leading cause of mortality in South Korea, contributing significantly to the national disease burden [1]. Given its high fatality rate and long-term health implications, effective risk stratification and comprehensive hospital mortality management are crucial for enhancing patient survival and ensuring quality care. In-hospital mortality serves as a crucial metric for evaluating healthcare quality, necessitating rigorous adjustment for patient-level characteristics, particularly comorbidity burden, given its profound influence on patient outcomes [2]. While not the primary admitting diagnosis, comorbidities are critical determinants of patient severity and significantly impact mortality risk, thus requiring careful consideration in analytical models [3].

The Charlson Comorbidity Index (CCI) is a widely used tool for quantifying comorbidity burden by assessing the presence and severity of pre-existing medical conditions [4]. However, its applicability has been questioned over time due to changes in the prevalence and impact of comorbid conditions [5]. To address these concerns, Quan et al. proposed a modified version of the CCI based on administrative health data, which has been validated and implemented in various settings, including Korea’s Hospital Standardized Mortality Ratio (HSMR) calculation [6,7]. Studies have shown that this modified CCI demonstrates improved predictive accuracy for mortality compared to the original CCI, particularly in specific patient populations and healthcare contexts [8,9,10]. Despite its broad application, this model does not incorporate data from Korean hospitalized patients, limiting its ability to reflect Korea’s inpatient population characteristics. Moreover, the fixed weighting system applies the same weights across all primary diagnoses and only accounts for 17 comorbidities, potentially excluding other significant mortality-related conditions.

Unlike traditional comorbidity indices that are limited to a predefined set of conditions, the Clinical Classifications Software (CCS), version 2015.1, groups ICD-coded diagnoses into clinically meaningful categories, allowing for a more comprehensive classification of diseases [11]. This approach enhances severity adjustment by capturing a broader spectrum of comorbid conditions, particularly in complex patient populations, and improves hospital mortality risk standardization. Studies have demonstrated that CCS-based risk adjustment outperforms traditional indices like the Charlson Comorbidity Index (CCI) in mortality prediction. As a result, several national healthcare systems, including those in the United States and the United Kingdom, have adopted CCS-based models for hospital benchmarking and quality assessment [11,12].

Efforts to refine comorbidity adjustment for mortality prediction in the United Kingdom led to the development of a UK-specific comorbidity measure, known as the Summary Hospital-level Mortality Indicator (SHMI) [13]. This model, derived from UK national discharge data, was designed to more accurately reflect the characteristics of UK hospital discharges by incorporating updated comorbidity weightings. It is now widely utilized in the development of hospital mortality prediction models and in evaluating hospital performance with enhanced precision and clinical applicability. This adaptation involved revising the list of included comorbidities and recalibrating their weights based on more recent population data. Nevertheless, it has the limitation of not fully accounting for all comorbidities that influence mortality, highlighting the ongoing need for further refinement in severity adjustment models.

In our current study, we aimed to develop severity adjustment methods for patients with acute cerebrovascular disease using Korean hospital discharge data. Specifically, we utilized the Korea Disease Control Agency’s Discharge Injury In-depth Survey Dataset to construct a comorbidity-based severity adjustment model. By focusing on acute cerebrovascular disease, we aim to develop a tailored severity adjustment tool that more precisely captures its clinical characteristics. Additionally, this study seeks to recalibrate the Charlson Comorbidity Index (CCI) while also generating a CCS-based comorbidity index that considers a broader range of diseases. Finally, we validated these models to ensure their effectiveness in mortality risk adjustment for Korean inpatients.

## 2. Materials and Methods

### 2.1. Data Source and Study Population

This study utilized 1,803,611 records from the Korea National Hospital Discharge In-depth Injury Survey (KNHDIS), which is conducted annually by the Korea Disease Control and Prevention Agency (KDCA) and contains nationwide administrative data on hospitalized patients from 2013 to 2022.

The study sample was extracted following the methodology for developing severity-adjusted mortality models and the Hospital Standardized Mortality Ratio (HSMR) assessments in Korea. From the dataset, 38,497 inpatient records were identified in which the primary diagnosis fell under the Acute Cerebrovascular Disease category, as classified by the Clinical Classifications Software (CCS) diagnosis group 109. Table 1 presents the International Classification of Diseases, 10th Revision (ICD-10) codes included in CCS category 109: Acute Cerebrovascular Disease.

Patients transferred to another hospital, cases with unknown or unspecified discharge status (8157 cases), and same-day discharges (256 cases) were excluded. The final study included 30,084 cases, which were used to recalculate the Charlson Comorbidity Index (CCI) and construct the CCS-based comorbidity index.

To validate the comorbidity indices, we followed the methodology from previous studies [12,14] by splitting the dataset into a training set, comprising records from 2013, 2015, 2017, 2019, and 2021 (14,888 cases), and a validation set, consisting of records from 2014, 2016, 2018, 2020, and 2022 (15,196 cases).

### 2.2. Variables

The dependent variable in this study was in-hospital mortality (death or survival). The independent variables, considered in the recalibration and validation of the Charlson Comorbidity Index (CCI) and the CCS-based comorbidity index, included sex, age, insurance type, surgical status, emergency admission status, and comorbidities, aligning with the variables used in the calculation of Korea’s Hospital Standardized Mortality Ratio (HSMR).

With respect to comorbidities, three distinct approaches were employed to assess their impact: (1) the CCI, the Charlson Comorbidity Index (CCI) currently used in Korea; (2) the m-CCI, a recalibrated version of the CCI developed specifically for this study; and (3) the m-CCS, a modified comorbidity index constructed using the Clinical Classifications Software (CCS) from the Agency for Healthcare Research and Quality (AHRQ), USA.

### 2.3. Statistical Analysis

For recalibrating the Charlson Comorbidity Index (CCI) and developing the CCS-based comorbidity index, Cox proportional hazards regression was used to estimate new comorbidity weights using survival analysis. We tested the proportional hazard assumption using **the Schoenfeld test.** For the m-CCI, hazard ratios (HRs) and 95% confidence intervals (CIs) were estimated while adjusting for sex, age, insurance type, surgical status, emergency admission status, and 17 CCI-defined comorbidities. Similarly, for the m-CCS, HRs and 95% CIs were estimated with adjustments for sex, age, insurance type, surgical status, emergency admission status, and 260 CCS-defined comorbidities, and normalized in the same manner.

As a result, the final recalibrated m-CCI and m-CCS comorbidity weights were established, and three models were developed based on these indices:

Model 1: Adjusted for age, gender, type of insurance, surgical status, emergency admission status, and baseline CCI.

Model 2: Adjusted for age, gender, type of insurance, surgical status, emergency admission status, and modified CCI (m-CCI).

Model 3: Adjusted for age, gender, type of insurance, surgical status, emergency admission status, and modified CCS (m-CCS).

The validity of the Charlson Comorbidity Index (CCI), as well as the recalibrated m-CCI and m-CCS indices, was assessed using traditional logistic regression and various machine learning approaches. For machine learning analysis, this study adopted the methodology proposed by Desai et al., employing Least Absolute Shrinkage and Selection Operator (LASSO) regression, the Classification and Regression Tree (CART), Random Forests, and the Gradient-Boosted Model (GBM) [15]. Additionally, an Artificial Neural Network (ANN), a fundamental deep learning model, was incorporated to further validate the model performance.

Model performance was assessed using the area under the receiver operating characteristic curve (AUC) with 95% confidence intervals (CIs) in both the training and validation datasets. The AUC values were used to assess the discriminative ability of the models and to ensure robustness in performance between the training and validation phases, ensuring consistency and reliability in predictive performance across datasets.

All statistical analyses were performed using SAS 9.4 for Windows (SAS Institute, Cary, NC, USA) and Python 3.10.0 for Windows (Python Software Foundation, Wilmington, DE, USA).

## 3. Results

### 3.1. Baseline Characteristics

The study included a total of 30,084 cases, which were divided into training (*n* = 14,888) and validation (*n* = 15,196) datasets. Table 2 summarizes the baseline characteristics of the study population. The cohort consisted of 57.0% males and 43.0% females, with similar distributions in the training (56.4% male) and validation (57.7% male) sets. The mean age of the overall population was 65.9 ± 14.64 years, and the majority of patients were covered by National Health Insurance (93.1%), followed by Medical Aid (6.3%). Most patients (86.0%) did not undergo surgery, and 80.5% were admitted through emergency departments, with similar distributions across the training and validation groups.

### 3.2. Recalibration of the Charlson Comorbidity Index (CCI) and the Development of the CCS-Based Comorbidity Index Using Survival Analysis

The comorbidity weights for the Charlson Comorbidity Index (CCI) were recalibrated using Cox proportional hazards regression based on a total of 30,084 cases. We confirmed a relationship between time and residuals in proportional hazard assumption using the Schoenfeld test (*p* = 0.291). Comorbidities with statistically significant hazard ratios (*p* < 0.05) and HR ≥ 1 were normalized by dividing them by the lowest HR, leading to the final recalibrated modified CCI (m-CCI) weights.

Among the comorbidities, moderate or severe liver disease (HR = 2.880, *p* < 0.0001) had the highest recalibrated m-CCI weights, indicating a strong association with in-hospital mortality (Table 3).

Among the 260 CCS-defined comorbidities, 55 comorbidities with a *p*-value ≤ 0.05 and a hazard ratio (HR) ≥ 1 were used to calculate the m-CCS weights. Leukemia (HR = 7.224) had the highest weight, followed by pancreatic disorder (HR = 5.315) and cardiac arrest and ventricular fibrillation (HR = 5.144), showing strong associations with in-hospital mortality. The model incorporated sex, age, insurance type, surgical status, and emergency admission status to ensure comprehensive risk adjustment. We confirmed a relationship between time and residuals in the proportional hazard assumption using the Schoenfeld test (*p* = 0.214) (Table 4).

### 3.3. Distribution of CCI, m-CCI, and m-CCS Comorbidity Indices

**Figure 1:**
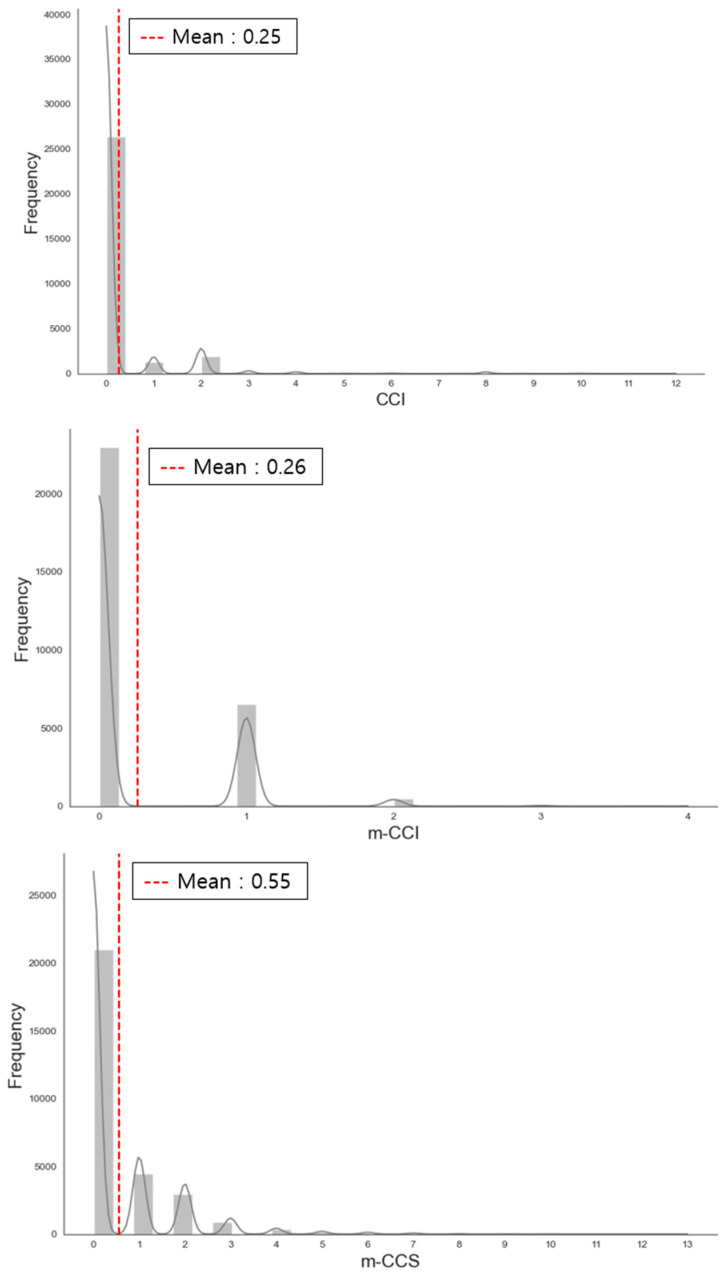
Distribution of CCI, m-CCI, and m-CCS comorbidity indices. CCI, the Charlson Comorbidity Index (CCI) currently used in Korea; m-CCI, a recalibrated version of the CCI developed specifically for this study; m-CCS, a modified comorbidity index constructed using the Clinical Classifications Software (CCS) from the Agency for Healthcare Research and Quality (AHRQ), Rockville, MD, USA.

The Charlson Comorbidity Index (CCI) has a mean value of 0.25, indicating that a large proportion of patients either have no comorbidities or very few, which aligns with a generally low comorbidity burden. The m-CCI, recalibrated based on hazard ratios, has a mean of 0.26, suggesting that the comorbidity burden is now more appropriately weighted and dispersed compared to the original CCI. The m-CCS has the highest mean value (0.55), indicating that it assigns higher overall comorbidity scores than both the CCI and m-CCI, reflecting a more comprehensive assessment of the comorbidity burden (Figure 1).

### 3.4. Validation of the Comorbidity Indices Using Machine Learning

**Figure 2:**
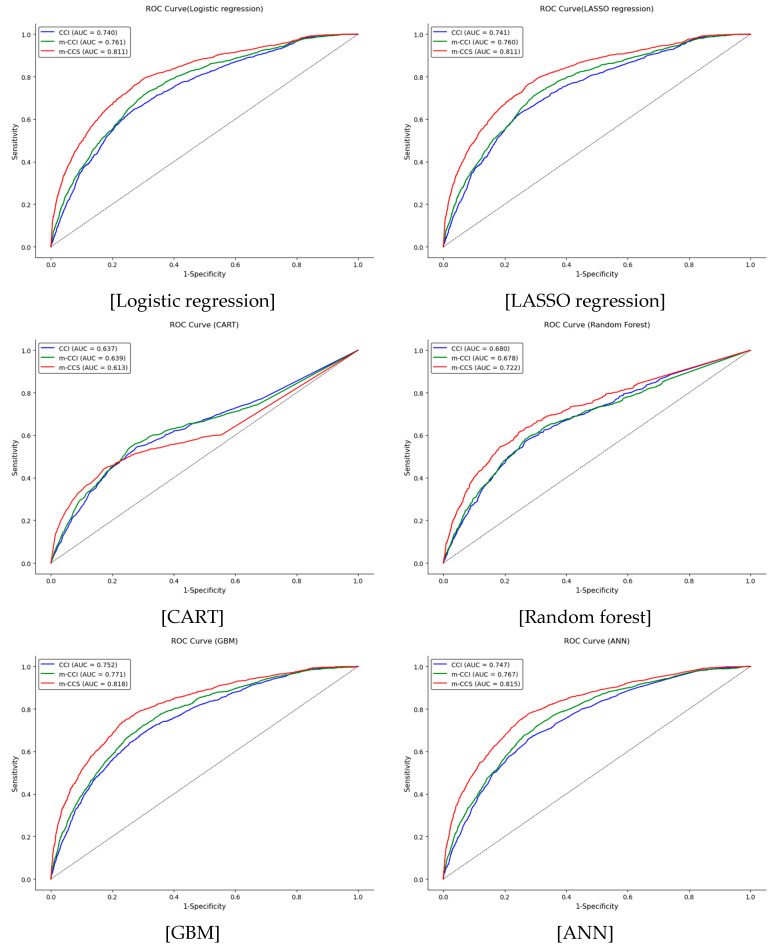
ROC curve. CCI, the Charlson Comorbidity Index (CCI) currently used in Korea; m-CCI, a recalibrated version of the CCI developed specifically for this study; m-CCS, a modified comorbidity index constructed using the Clinical Classifications Software (CCS) from the Agency for Healthcare Research and Quality (AHRQ), USA; AUC, area under the ROC curve; LASSO, Least Absolute Shrinkage and Selection Operator; CART, Classification and Regression Tree; GBM, Gradient Boosting Machine; ANN, Artificial Neural Network; ROC, receiver operating characteristic.

Table 5 presents the area under the ROC curve (AUC) values and 95% confidence intervals (CIs) for different models validated on training (*n* = 14,888) and validation (*n* = 15,196) datasets. The m-CCS comorbidity index consistently demonstrated the highest predictive performance across all models, achieving the highest AUC values in both training and validation datasets. Among the models, GBM (AUC = 0.835 in training, 0.818 in validation) and ANN (AUC = 0.830 in training, 0.815 in validation) showed the highest overall AUC values, demonstrating their superiority over traditional regression models, while CART performed the weakest, with lower AUC values and less differentiation among the three indices. The m-CCI outperformed the original CCI, indicating that the recalibrated index enhances risk prediction accuracy (Figure 2).

## 4. Discussion

In this study, we developed severity adjustment methods for acute cerebrovascular disease patients using data from the Korea National Hospital Discharge In-depth Injury Survey (KNHDIS). By constructing a comorbidity-based severity adjustment model, we aimed to enhance the precision of risk adjustment for Korean inpatients. Our approach involved recalibrating the Charlson Comorbidity Index (CCI) and generating a CCS-based comorbidity index that incorporates a broader spectrum of diseases. Through rigorous validation, we demonstrated the effectiveness of these models in mortality risk adjustment, ensuring their applicability in assessing hospital performance and patient outcomes in the Korean healthcare setting.

Recent studies have underscored the necessity of refining comorbidity indices to enhance the accuracy of mortality risk adjustment [16,17]. The findings of this study have demonstrated the robust predictive validity of the recalibrated modified Charlson Comorbidity Index (m-CCI) and the newly developed comorbidity index based on Clinical Classifications Software (m-CCS). These indices are particularly effective in identifying high-risk conditions among patients with acute cerebrovascular disease, including moderate or severe renal disease, malignancies, leukemia, pancreatic disorders, and cardiac arrest. These results are particularly crucial for patients with acute cerebrovascular disease, for whom comorbidity burden plays a critical role in clinical outcomes.

From the distribution pattern of the comorbidity index, we revealed a more pronounced right-skewed distribution in the m-CCS model compared to CCI and m-CCI. This skewness indicates that while most patients have low comorbidity scores, a subset with severe comorbidities receives significantly higher scores. The greater skewness in the m-CCS model suggests its enhanced ability to distinguish patients with higher comorbidity burdens, assigning more precise and elevated scores. As a result, m-CCS captures a broader spectrum of disease severity, offering a more comprehensive assessment of comorbidity burden than CCI and m-CCI.

Furthermore, by utilizing the CCS-based comorbidity index (m-CCS), this study developed a tailored severity adjustment tool that accurately reflects the unique clinical characteristics of acute cerebrovascular disease, as demonstrated by its consistently highest AUC values. Unlike traditional indices, the m-CCS model allows for a more comprehensive evaluation of all disease categories, enabling a more refined risk adjustment approach. This advancement enhances the precision of severity assessment, thereby improving mortality risk prediction, hospital performance evaluation, and data-driven clinical decision-making in Korean healthcare settings.

By incorporating key demographic and clinical variables—including sex, age, insurance type, surgical status, and emergency admission status—this study contributes to a more refined risk adjustment methodology specifically for acute cerebrovascular disease. However, future research should focus on developing tailored severity adjustment tools that reflect the unique characteristics of various diseases, ensuring more precise risk stratification across different patient populations. Additionally, clearly identifying comorbidities that significantly impact mortality is crucial for guiding clinical decision-making and optimizing the management of high-risk patients. By providing clinicians with more accurate comorbidity-based risk assessments, these tools can support improved patient care and more effective interventions for critically ill individuals.

This study employed a combination of statistical, machine learning, and deep learning techniques to develop and validate severity adjustment methods. Given that severity-adjusted ratios are publicly disclosed at the national level, ensuring their accuracy and reliability through high-performance predictive models is crucial [14]. However, a key limitation of advanced analytical techniques, particularly deep learning, is their lack of interpretability, making it difficult to understand how specific outcomes are generated. Since clinicians play a vital role in patient care and mortality management, it is essential to provide transparent, evidence-based insights into the comorbidities that significantly impact mortality for each primary diagnosis. Enhancing the transparency and clinical applicability of risk adjustment models will enable healthcare professionals to integrate these tools into evidence-based practice, ultimately improving patient management and healthcare decision-making.

Despite the strengths of this study, several limitations should be acknowledged. First, this study was developed using discharge patient data exclusively from Korea to establish a Korean-specific severity adjustment method. Consequently, the results may reflect characteristics unique to the Korean healthcare system and may not be directly applicable to other populations. In addition, as the dataset lacked hospital-level and socioeconomic variables, future research should incorporate these factors to enhance the model’s generalizability and applicability across different settings. Second, this study used an alternating-year split method based on prior research, but this approach may introduce temporal bias due to changes in clinical practices and patient characteristics over time. Moreover, k-fold cross-validation or resampling methods were not used to assess generalizability or reduce overfitting, which we acknowledge as a limitation for future improvement. The severity adjustment tool was developed using statistical analysis, which, while robust, does not fully incorporate clinical expertise from physicians. Integrating clinical insights is necessary to enhance its practical utility. Additionally, due to sample size constraints, more advanced artificial intelligence techniques, such as deep learning, could not be fully applied in this study. Future research should address these limitations by expanding data availability, incorporating a broader range of AI methodologies, and adopting approaches that reflect the unique characteristics of each country to refine severity adjustment models. Such improvements will contribute to more precise risk stratification, better resource allocation, and an overall enhancement in healthcare system efficiency.

## Figures and Tables

**Table 1:** Definition of Acute Cerebrovascular.

CCS Category	Diagnosis	ICD-10 Code
109	Acute Cerebrovascular Disease	G46.0 *~G46.8 *, I60.0~I60.9, I61.0~I61.6, I61.8, I61.9, I62.0, I62.1, I62.9, I63.0~I63.6, I63.8, I63.9, I64, I66.0~I66.4, I66.8, I66.9

CCS, Clinical Classifications Software; ICD, International Classification of Diseases; * the asterisk code is applied when the condition is considered a manifestation of an underlying disease; ~ the tilde symbol is used to denote a continuous range of ICD-10 codes. For example, “G46.0 *~G46.8 *” encompasses every code from G46.0 * through G46.8 *.

**Table 2:** Baseline characteristics of study population.

Characteristics	Total (*n* = 30,084)	Training (*n* = 14,888)	Validation (*n* = 15,196)
Sex			
Male	17,159 (57.0)	8390 (56.4)	8769 (57.7)
Female	12,925 (43.0)	6498 (43.6)	6427 (42.3)
Age, mean (SD), years	65.9 (14.64)	65.8 ± 14.65	65.9 ± 14.62
Insurance type			
National Health Insurance	28,014 (93.1)	13,854 (93.1)	14,160 (93.2)
Medical Aid	1899 (6.3)	955 (6.4)	944 (6.2)
Others	171 (0.6)	79 (0.5)	92 (0.6)
Surgical Status			
No	25,886 (86.0)	12,843 (86.3)	13,043 (85.8)
Yes	4198 (14.0)	2045 (13.7)	2153 (14.2)
Emergency admission			
No	5858 (19.5)	2868 (19.3)	2990 (19.7)
Yes	24,226 (80.5)	12,020 (80.7)	12,206 (80.3)

**Table 3:** Recalibration of the Charlson Comorbidity Index (CCI).

Comorbidity	Total (*n* = 30,084)					
	Parameter Estimate	Standard Error	Hazard Ratio	*p* Value	CCI	m-CCI *
Myocardial infarct	0.185	0.180	1.203	0.303	-	-
Congestive heart failure	0.547	0.110	1.729	<0.0001	2	1
Peripheral vascular disease	0.061	0.207	1.063	0.767	-	-
Cerebrovascular disease	0.504	0.046	1.655	<0.0001	-	1
Dementia	−1.044	0.168	0.352	<0.0001	2	-
Chronic pulmonary disease	−0.064	0.156	0.938	0.682	1	-
Connective tissue disease	0.214	0.280	1.239	0.444	1	-
Ulcer disease	−0.302	0.222	0.739	0.174	-	-
Mild liver disease	0.440	0.137	1.553	0.001	2	1
Diabetes with end organ damage	−0.212	0.144	0.809	0.014	1	-
Diabetes	−0.335	0.061	0.715	<0.0001	-	-
Hemiplegia	−1.753	0.103	0.173	<0.0001	2	-
Moderate or severe renal disease	0.630	0.085	1.878	<0.0001	1	1
Any tumor, leukemia, lymphoma	0.798	0.112	2.222	<0.0001	2	1
Moderate or severe liver disease	1.058	0.295	2.880	<0.0001	4	2
Metastatic solid tumor	0.090	0.205	1.094	0.663	6	-
AIDS	0.689	1.001	1.991	0.491	4	-

* The model incorporates sex, age, insurance type, surgical status, emergency admission status, and all 17 CCI-defined comorbidities, with comorbidity weights recalibrated for CCI comorbidities with a *p*-value of ≤0.05 and a hazard ratio (HR) of ≥1.

**Table 4:** Development of the CCS-based comorbidity index.

Comorbidity	Total (*n* = 30,084)				
	Parameter Estimate	Standard Error	Hazard Ratio	*p* Value	m-CCS *
Other and ill-defined cerebrovascular disease	0.211	0.080	1.235	0.009	1
Pneumonia	0.236	0.080	1.266	0.003	1
Immunizations and screening for infectious disease	0.285	0.117	1.330	0.015	1
Residual codes; unclassified	0.307	0.069	1.359	<0.0001	1
Respiratory failure; insufficiency; arrest	0.430	0.173	1.537	0.013	1
Other screening for suspected conditions	0.445	0.132	1.561	0.001	1
Coagulation and hemorrhagic disorders	0.458	0.165	1.580	0.005	1
Congestive heart failure; nonhypertensive	0.464	0.125	1.590	<0.0001	1
septicemia	0.531	0.106	1.701	<0.0001	1
Aspiration pneumonitis; food/vomitus	0.565	0.090	1.759	<0.0001	1
Chronic renal failure	0.572	0.089	1.772	<0.0001	1
Acute myocardial infarction	0.657	0.208	1.930	0.002	2
Acute and unspecified renal failure	0.774	0.094	2.168	<0.0001	2
Acute cerebrovascular disease	0.823	0.049	2.277	<0.0001	2
Cancer of liver and intrahepatic bile duct	0.956	0.217	2.601	<0.0001	2
Cancer of bronchus; lung	0.985	0.228	2.677	<0.0001	2
Shock	1.016	0.385	2.761	0.008	2
Non-Hodgkin’s lymphoma	1.111	0.506	3.038	0.028	2
Other mental conditions	1.203	0.310	3.329	<0.0001	3
Cancer of cervix	1.420	0.504	4.137	0.005	3
Systemic lupus erythematosus and connective tissue disorders	1.494	0.336	4.457	<0.0001	4
Coma; stupor; and brain damage	1.519	0.193	4.569	<0.0001	4
Nervous system congenital anomalies	1.536	0.580	4.648	0.008	4
Cardiac arrest and ventricular fibrillation	1.638	0.108	5.144	<0.0001	4
Pancreatic disorders	1.671	0.357	5.316	<0.0001	4
Leukemias	1.977	0.216	7.224	<0.0001	6

* The model incorporates sex, age, insurance type, surgical status, emergency admission status, and all 260 CCS-defined comorbidities, with comorbidity weights calculated separately for 26 CCS comorbidities selected through a stepwise method, each having a *p*-value < 0.05 and a hazard ratio (HR) ≥ 1.

**Table 5:** Validation of the comorbidity indices using machine learning.

	Training (*n* = 14,888)			Validation (*n* = 15,196)		
	AUC	95% CI		AUC	95% CI	
		Lower	High		Lower	High
Logistic regression						
CCI	0.744	0.730	0.759	0.740	0.725	0.754
m-CCI	0.765	0.752	0.778	0.761	0.746	0.774
m-CCS	0.811	0.798	0.824	0.811	0.798	0.825
LASSO regression						
CCI	0.743	0.729	0.758	0.741	0.725	0.755
m-CCI	0.764	0.752	0.778	0.760	0.746	0.774
m-CCS	0.811	0.798	0.824	0.811	0.798	0.825
CART						
CCI	0.659	0.650	0.669	0.637	0.620	0.654
m-CCI	0.673	0.664	0.682	0.639	0.620	0.658
m-CCS	0.721	0.714	0.729	0.613	0.594	0.633
Random forest						
CCI	0.652	0.642	0.663	0.680	0.664	0.696
m-CCI	0.667	0.658	0.677	0.678	0.661	0.695
m-CCS	0.713	0.706	0.721	0.722	0.705	0.739
GBM						
CCI	0.773	0.760	0.788	0.752	0.737	0.766
m-CCI	0.792	0.780	0.805	0.771	0.757	0.785
m-CCS	0.835	0.822	0.847	0.818	0.806	0.831
ANN						
CCI	0.773	0.760	0.787	0.747	0.733	0.761
m-CCI	0.789	0.776	0.801	0.767	0.752	0.780
m-CCS	0.830	0.818	0.843	0.815	0.802	0.828

CCI, the Charlson Comorbidity Index (CCI) currently used in Korea; m-CCI, a recalibrated version of the CCI developed specifically for this study; m-CCS, a modified comorbidity index constructed using the Clinical Classifications Software (CCS) from the Agency for Healthcare Research and Quality (AHRQ), USA; AUC, area under the ROC curve; CI, confidence interval; LASSO, Least Absolute Shrinkage and Selection Operator; CART, Classification and Regression Tree; GBM, Gradient Boosting Machine; ANN, Artificial Neural Network.

## Data Availability

The datasets analysed during the current study are available from the Korea Disease Control and Prevention Agency on reasonable request [http://www.kdca.go.kr/contents.es?mid=a20303010502, accessed on 2 December 2024].
